# Reviving the Grasp: A Case Report on a Pioneering Approach to Managing Crush Syndrome and Unveiling the Occult Compartment Syndrome

**DOI:** 10.7759/cureus.55370

**Published:** 2024-03-02

**Authors:** Hoe Teong Kee, Mohd Shahril Jaapar, Manohar Arumugam, Firdati Mohamed Saaid, Collin Looi, Fahrudin Che-Hamzah

**Affiliations:** 1 Orthopaedics and Traumatology, Hospital Sultan Abdul Aziz Shah, Serdang, MYS; 2 Hand and Microsurgery Unit, Hospital Sultan Abdul Aziz Shah, Serdang, MYS

**Keywords:** hand and microsurgery, traumatic brachial plexus injury, emergency fasciotomy, hand compartment syndrome, crush syndrome

## Abstract

A crush injury results directly from a crushing force, while crush syndrome, or traumatic rhabdomyolysis, manifests as systemic consequences stemming from the breakdown of muscle cells. Hand crush injuries present intricate challenges involving damage to multiple structures, tissue loss, and potential digit amputation, often caused by high-energy trauma. Each case demands a unique management plan, with the critical decision between limb salvage and amputation. Early intervention to restore vascularity is pivotal for preserving hand function. The complexity is heightened by the occult compartment syndrome, characterized by increased pressure causing neurovascular compromise without external signs. A patient with an insensate limb due to ipsilateral pan brachial plexus injury (BPI) adds an additional layer of complexity to the management journey, emphasizing the need for a multidisciplinary approach. This case is unique and underscores the importance of prioritizing reconstruction, identifying crush syndrome and the occult compartment syndrome, and employing a strategic, decisive approach that includes various surgical techniques for optimal outcomes in complex hand injuries.

## Introduction

The hand is a complex structure comprising several tissues (skin, nerves, blood vessels, tendons, bones joints, and intrinsic muscles) that are closely packed in a small space [1]. Hand and wrist injuries are one of the major causes of functional disability worldwide. A crush injury of the hand indicates an injury to multiple structures of the hand, jeopardizing its function and viability. It may include bone, soft tissue, and neurovascular injuries [1].

Crush trauma to the extremities, even when not involving vital organs, poses a potential threat to life. The term "crush injury" specifically refers to damage caused directly by a crushing force [1]. By contrast, crush syndrome, also recognized as traumatic rhabdomyolysis, manifests as a systemic breakdown of muscle cells, releasing their contents into the circulation, and is a well-known complication of crush injuries that can lead to acute kidney injury (AKI), representing a potentially life-threatening scenario that can be both prevented and reversed [2].

The initial documentation of crush syndrome dates back to 1941 when Bywaters and Beall observed its occurrence after the Battle of London [3]. Patients initially rescued from debris appeared uninjured, but subsequently developed progressive limb swelling and shock and succumbed to renal failure within days [3]. Postmortem examinations revealed muscle necrosis and brown pigment casts in renal tubules [3]. While crush injuries are commonly associated with natural disasters, such as earthquakes, emergency physicians often encounter the syndrome in patients involved in motor vehicle collisions, particularly those subjected to prolonged extrication, and victims of assault [4].

Acute compartment syndrome (ACS) is a recognized and concerning complication that may arise following traumatic injuries [5]. If not promptly addressed, this syndrome can lead to significant and potentially limb-threatening consequences [5]. The initial documentation of ACS dates back to 1881 when Richard von Volkmann first described it, and it is defined by elevated pressure within a restricted fascial space [6]. Severe pain disproportionate to the examination findings typically manifests as the initial symptom. Descriptions of this pain often include terms, such as severe, deep, burning, and aggravated by passive stretching of the affected compartment. Regrettably, several symptoms including severe pain, pain exacerbated by passive stretch, paresthesia, and paresis exhibit poor sensitivity for diagnosis, making their absence insufficient to rule out the condition. Paresthesias, sensory deficits, and focal motor weakness may emerge later in the diagnostic process, potentially indicating a more unfavorable prognosis. [7].

Occult compartment syndrome is characterized by elevated pressure within a confined anatomical space, leading to neurovascular compromise without discernible external indicators [8]. This condition typically manifests in patients with an insensate limb and is frequently observed in unconscious individuals [8]. The absence of overt external signs poses a significant challenge to the early detection of occult compartment syndrome, emphasizing the crucial need for clinical vigilance and a thorough evaluation. The objective of managing ACS is to alleviate the elevated intracompartmental pressure (ICP), which poses a threat to the limb or affected area. Treatment should be promptly initiated if there is any uncertainty, given the potential for catastrophic outcomes [7].

The diagnosis of ACS necessitates the evaluation of ICP, typically achieved through direct and invasive monitoring [9]. Among the most prevalent methods for assessing ICP is the solid-state transducer intracompartmental catheter (STIC) device, such as the Stryker Intra-Compartmental Pressure Monitor System [9]. This device utilizes a pressure transducer to directly measure the pressure within the catheter lumen [9]. Studies have shown the STIC device to possess high accuracy, with a sensitivity of 94% and a specificity of 98% [9]. If a fracture is present, it is recommended that the provider position the catheter within 5 cm of the fracture level. However, caution should be exercised to avoid placing the transducer directly within the fracture site, as doing so may result in falsely elevated ICP readings. One study reported that continuous ICP monitoring, combined with a ΔP of 30 mm Hg or less, alongside clinical symptoms, yielded a sensitivity of 61% and specificity of 97% for diagnosing ACS [10].

This case is unique, involving both crush syndrome and occult compartment syndrome in an insensate limb. Its aim is to increase awareness among physicians, prompting timely management to improve outcomes and facilitate successful limb-saving surgeries.

## Case presentation

A 30-year-old gentleman, who is right-hand dominant and has a past medical history of a successfully excised benign brain tumor eight years ago, with well-managed epilepsy on tab epilim 800 mg BD, presented within 12 hours of sustaining an injury. As a motorbike rider involved in a motor vehicle accident on the highway, he experienced a near-total amputation of his left thumb and a severe crush injury to his left hand after colliding with a divider attributed to a micro-sleep episode. The affected hand was contaminated with grass and sand. The patient was referred to our hospital with hand and microsurgery services following an extensive crush injury to the hand.

His Glasgow coma scale was 15/15. Vital signs revealed tachypnea with a respiratory rate of 24 breaths per minute, blood pressure measuring 100/70, tachycardia with a heart rate of 112 beats per minute, and the absence of fever, indicative of the patient being in hypovolemic shock. Blood pressure improved following initial fluid resuscitation with crystalloids.

**Figure 1:**
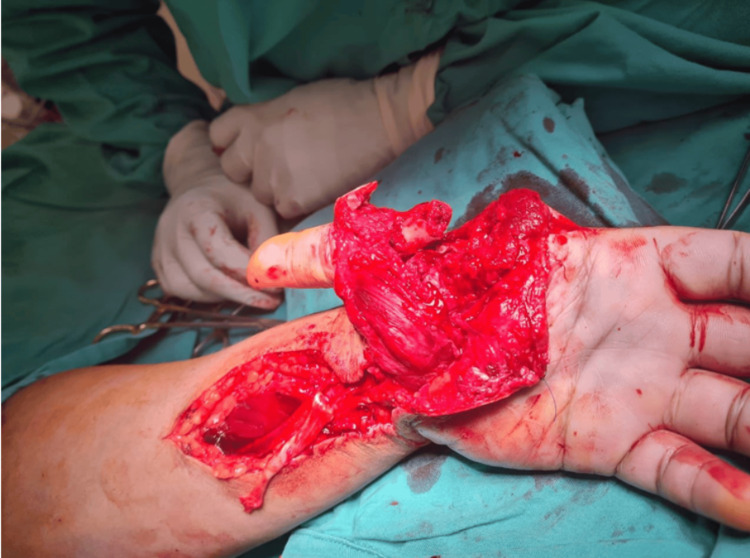
Clinical picture reveals a nearly circumferential lacerated wound over the left thenar eminence, extending to the dorsal first web space, accompanied by a dangling left thumb and severe soft tissue swelling in the left hand and forearm.

Upon local examination, a near circumferential lacerated wound was observed over the left thenar eminence, extending to the dorsal first web space, with a dangling left thumb and severe soft tissue swelling in the left hand and forearm (Figure 1). The volar compartment of the left hand and forearm were tense, with intervening tissue bridges showing prolonged capillary refill and poor oxygen saturation via pulse oximetry in all fingers. No movement was visible over the left shoulder joint and below, and the neurological examination revealed an insensate limb from the C5 to T1 dermatome. The passive stretch test was negative, and the patient did not experience pain over the left upper limb. The examination of the cervical region was unremarkable, and no ecchymoses were observed over the supraclavicular region. The diagnosis of acute compartment syndrome is based solely on clinical suspicion, leading to immediate preparation for urgent fasciotomy.

**Figure 2:**
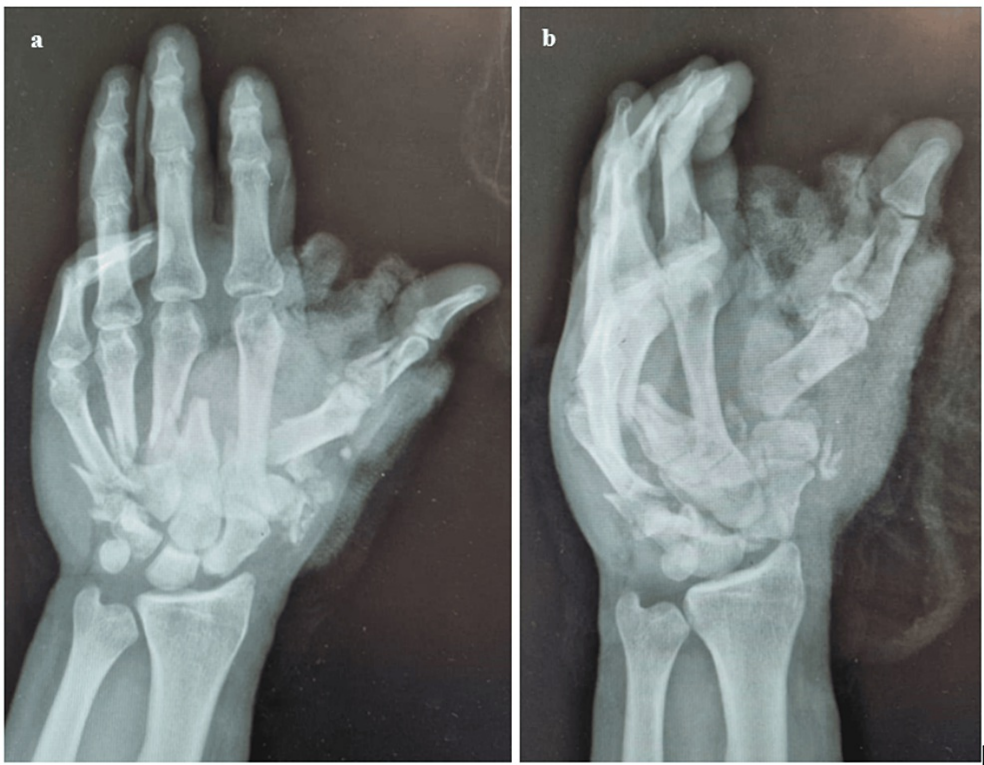
Radiographs of the left hand and wrist revealed a comminuted fracture of the first to fifth metacarpal bones along with carpal row dissociation a) Left-hand radiograph (posteroanterior view). b) Left-hand radiograph (oblique view).

Further examination unveiled additional injuries, including a closed complete post-ganglionic pan-brachial plexus injury (BPI) with an insensate limb, acute compartment syndrome of the volar compartment of the hand and forearm, a frontal laceration, and subconjunctival hemorrhage in the left eye. Radiographs of the left hand and wrist revealed a comminuted fracture of the first to fifth metacarpal bones along with carpal row dissociation (Figure 2a-2b). These injuries necessitate emergency wound debridement, irrigation, exploration of the artery, hand and forearm fasciotomy, and stabilization of fractures. Empirical antibiotics, including intravenous cephalosporin, nitroimidazole, and aminoglycoside, were initiated according to our local antibiotic protocol. Arrangements were made promptly for an immediate transfer to the operating room. Blood parameters showed elevated levels of potassium, aspartate transaminase (AST), lactate dehydrogenase (LDH), and creatinine kinase, indicative of a diagnosis of crush syndrome (Table 1). A urinary catheter was inserted, revealing clear urine, and aggressive fluid resuscitation with six liters of normal saline per day was initiated.

**Table 1:** Trend of blood results Hb: hemoglobin; TWBC: total white blood count; K^+^: potassium; Na^+^: sodium; CK: creatinine kinase; LDH: lactate dehydrogenase; AST: aspartate transaminase; CRP: C-reactive protein; ESR: erythrocyte sedimentation rate ↑ : increasing trend, ↔ : static, ↓ : reducing trend, ↑↑ : elevated, ↓↓ : reduced, - : not applicable

Parameter (units)	Results on admission	Results 24 hours after initial debridement and resuscitation	Results 72 hours after initial debridement and resuscitation	Results one week after initial debridement and resuscitation	Results before soft tissue closure	Reference range
Hb (g/L)	10.4	10.8 ↔	-	11.9 ↔	12.8 ↔	13.0-17.0
TWBC (x10^9^/L)	10.74	9.15 ↔	-	10.96 ↔	5.07 ↔	4.00-10.00
Platelet count (x10^9^/L)	139 ↓↓	230 ↑	-	324 ↑	369 ↔	150-410
Na^+^(mmol/L)	134	138 ↔	136 ↔	136 ↔	136 ↔	136-145
K^+ ^(mmol/L)	5.2 ↑↑	4.1 ↓	4.1 ↔	4.7 ↔	4.1 ↔	3.5-5.1
Urea (mmol/L)	6.4	3.7 ↓	3.7 ↔	3.6 ↔	5.5 ↔	3.2-7.4
Creatinine (micromoles/L)	92	69 ↓	64 ↔	69 ↔	73 ↔	63.6-110.5
CK (U/L)	2314 ↑↑	1502 ↓	851 ↓	-	-	30-200
LDH (U/L)	360 ↑↑	163.0 ↓	188 ↔	-	-	125-220
AST (U/L)	202 ↑↑	-	-	-	-	10-45
CRP (mg/L)	100 ↑↑	-	109 ↔	-	10 ↓	1-5
ESR (mm/hour)	-	-	91	-	-	

**Figure 3:**
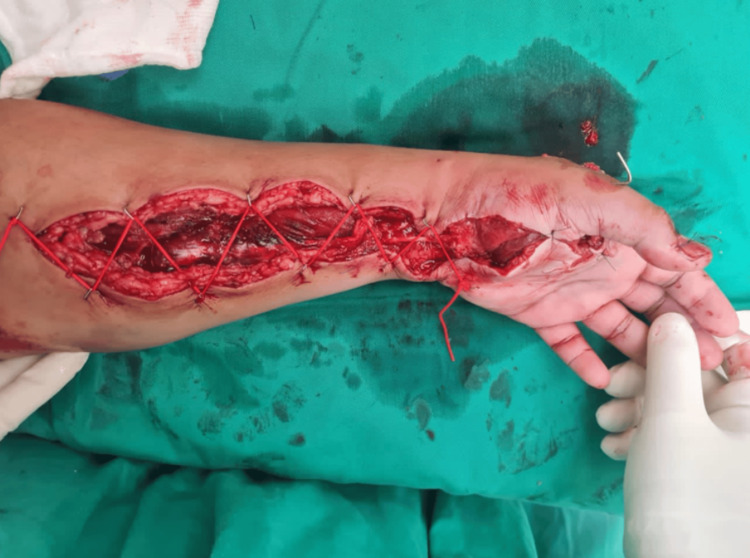
Intraoperative clinical picture reveals marked swelling of volar compartment muscles in the forearm successfully decompressed with full-length volar fasciotomy wound, tagged using a “shoelace technique” to facilitate subsequent wound closure. Wound closure utilized a thenar eminence skin flap to envelop underlying structures, the thenar eminence muscles, and repaired flexor pollicis longus tendon. Fractures were temporarily stabilized using K-wires, and finger posture and alignment were restored to a natural position without finger rotation.

Radiographs revealed metacarpal fractures, necessitating stabilization. However, the contamination with dirt poses an infection risk, discouraging the use of plates. Temporary fracture reduction and stabilization with K-wires were considered for their easy removal in the case of deep infection and subsequent definitive fixation. The intraoperative findings revealed a type IIc severe crush injury to the left hand based on Tulipan et al. [11], with near-total amputation of the left thumb highlighting the critical need for salvage surgery. Meticulous surgical technique, including magnification with loupes, was essential. Identification of the princeps pollicis artery and dorsal branch of the radial artery were integral components of the procedure. In addition, there was an open comminuted fracture involving the left first to fifth metacarpal bones and the proximal phalanx of the left thumb. Proximal carpal row dissociation was noted secondary to ligamentous disruption, along with the hand and forearm acute compartment syndrome. Other findings included transection of the flexor carpi radialis, flexor pollicis longus, and princeps pollicis artery contusion and thrombosis (Figure 3).

**Figure 4:**
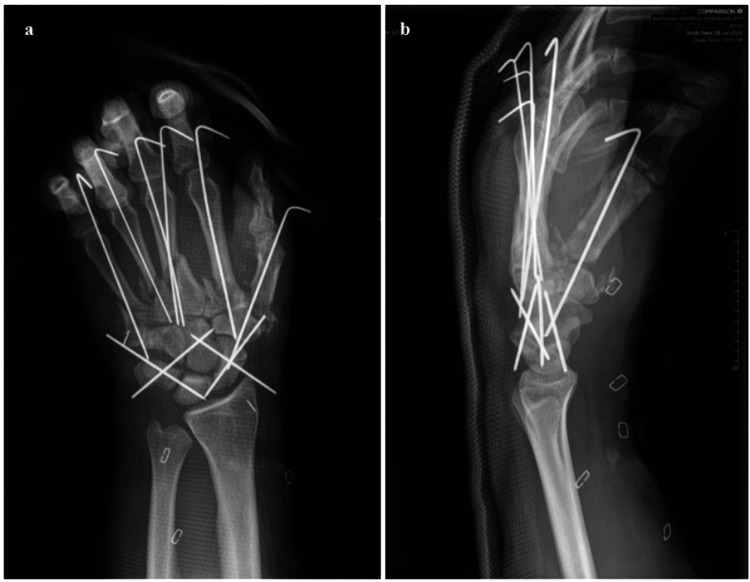
Postoperative radiographs of the left hand reveal successful stabilization of hand fractures using multiple K-wires during the primary debridement procedure. Fracture reduction and stabilization focused on restoration of the knuckle height and correcting fracture angulation, rotation, and shortening. Carpal row dissociation was addressed by employing four K-wires arranged in a diamond-shaped configuration, effectively securing the proximal and distal carpal rows while restoring Gigula’s carpal arcs. a) Left hand radiograph (posteroanterior view). b) Left hand radiograph (lateral view)

Fracture stabilization involved the strategic use of K-wires (Figure 4a-4b) to mitigate the heightened risk of infection. Debridement of the fracture ends was performed. K-wires were inserted in the coronal plane, intentionally left longer to emerge at the edge of the remaining skin flap. This approach facilitated uncomplicated removal without necessitating flap detachment in the event of infection. The patient's treatment regimen included subcutaneous administration of clexane 40 mg once daily. Continuous observation using a porch lamp was implemented to mitigate the risk of thrombosis or vasoconstriction following the intima injury.

**Figure 5:**
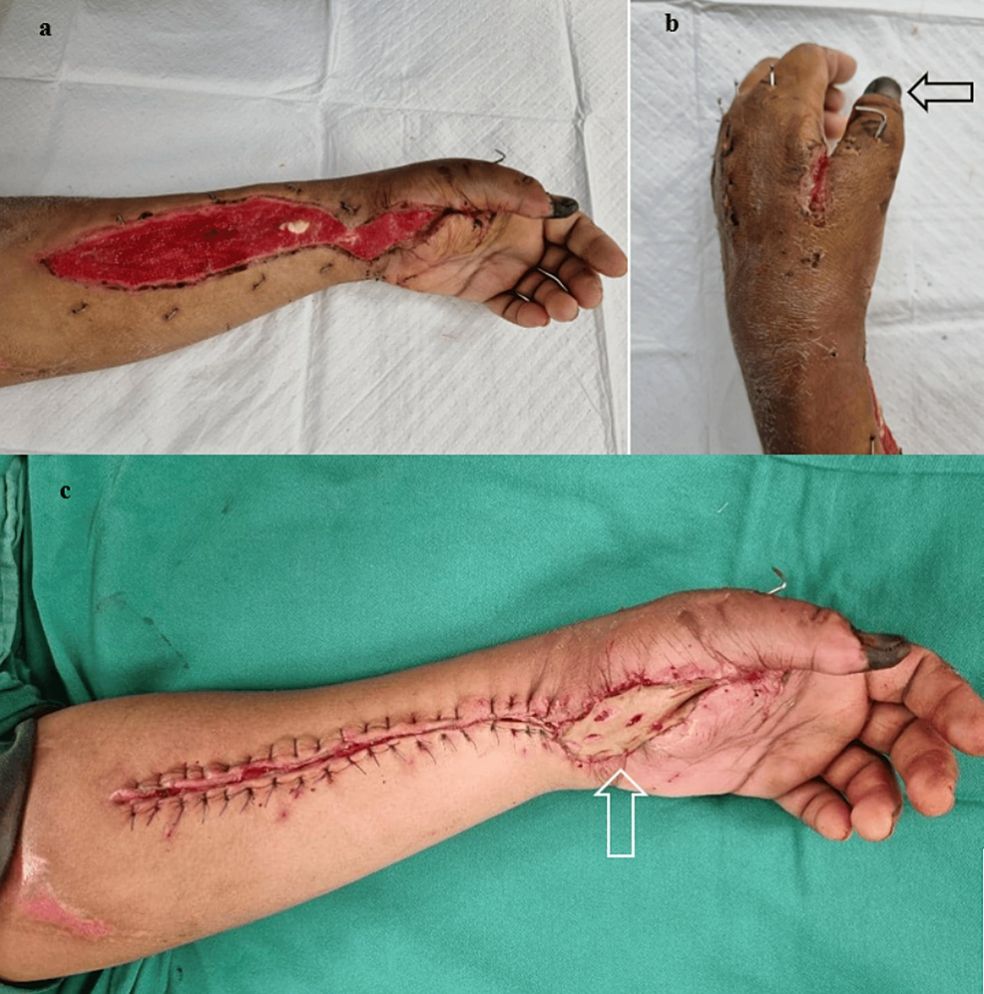
Clinical pictures of the left forearm a) Clinical image displays a well-vascularized wound bed, featuring healthy granulation tissue covering the volar aspect of the left forearm, the flexor crease of the left wrist, and the thenar eminence. b) Clinical image exhibits the healing of the left dorsal first web space wound through secondary intention, while the distinct line of demarcation indicates dry gangrene of the left thumb (black arrow), which has been intentionally left undisturbed. c) Intraoperative clinical picture reveals the secondary suturing of the left forearm fasciotomy wound devoid of soft tissue tension. In addition, a full-thickness skin graft was applied to cover the left wrist flexor crease and the thenar eminence, preventing the occurrence of contractures (white arrow).

Secondary suturing of the left forearm fasciotomy wound and full-thickness skin graft harvested from the right inguinal region were applied toward thenar eminence three-week post-primary debridement surgery. The donor side was closed primarily with Dafilon 4/0 suture (B. Braun®, United Kingdom) via the vertical mattress method. A full-thickness skin graft measuring 6 cm long and 5 cm wide was harvested, prepared, and sutured to a well-prepared vascularized thenar eminence wound bed with monosyn 4/0 sutures (B. Braun®, United Kingdom) via a simple interrupted method (Figure 5a-5c).

The patient underwent physiotherapy and postoperative rehabilitation. Regular monitoring of blood parameters, including the renal profile, creatinine kinase, lactate dehydrogenase, and C-reactive protein (CRP), showed a decreasing trend (Table 1). Subsequently, fluid resuscitation was withheld one week after the initial debridement surgery.

**Figure 6:**
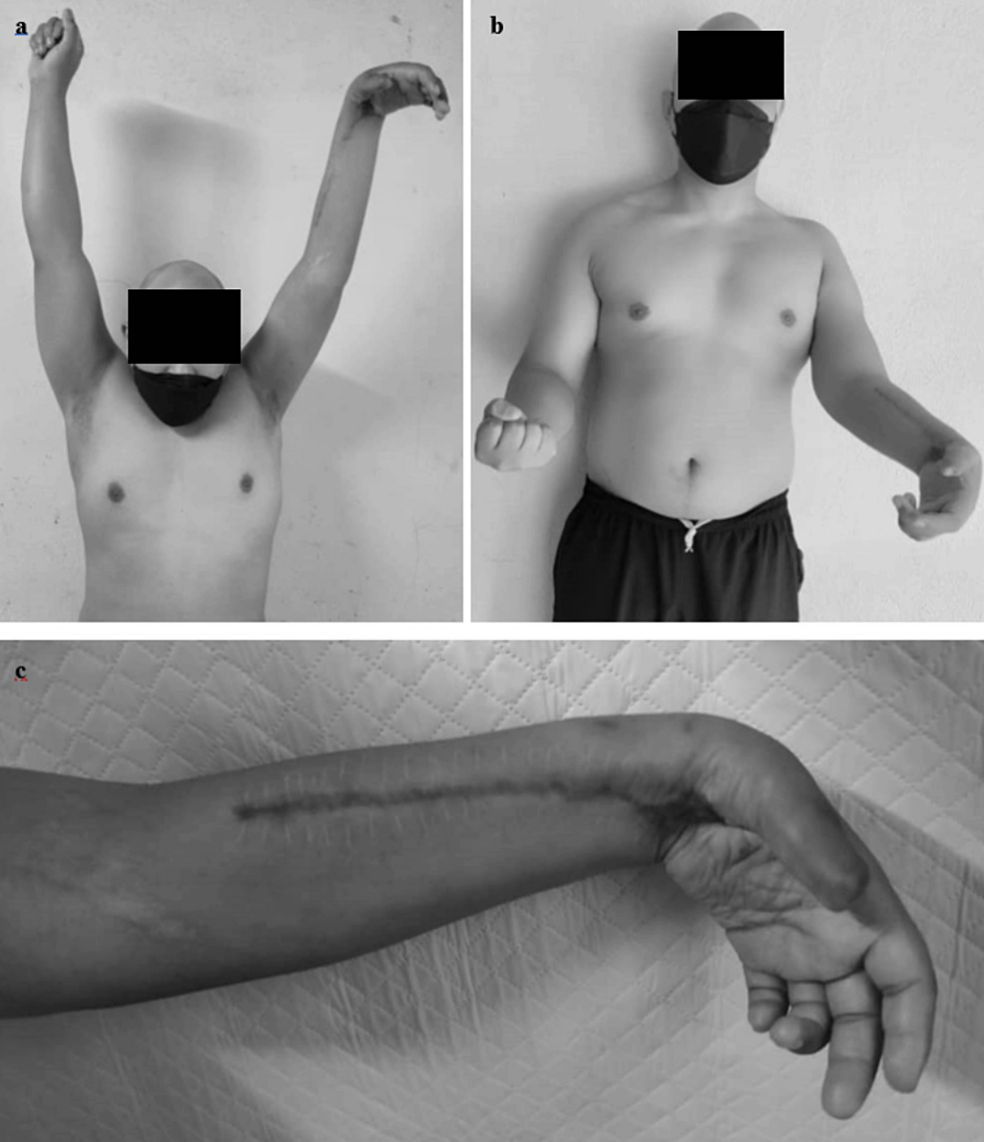
Clinical images depicting the clinical recovery of the left pan-brachial plexus injury. a) The clinical picture demonstrates that the patient can achieve nearly full left shoulder abduction compared to the contralateral side. b) The clinical picture reveals that the patient can perform active elbow flexion with a Medical Research Council (MRC) muscle power scale of grade 4. c) The clinical picture indicates the ability to perform wrist flexion with an MRC muscle power scale of grade 3.

Considering that the patient also experienced an associated injury involving a complete post-ganglionic left pan-BPI, it became crucial to preserve joint flexibility in order to prevent contractures, especially due to the anticipated extended recovery period associated with the BPI. At six months post-trauma, clinical examinations revealed the progression of the Tinel sign, evidence of deep muscle tenderness, and a significant improvement in shoulder abduction, elbow, and wrist flexion (Figure 6a-6c). These findings collectively point toward a progressive recovery of the BPI.

**Figure 7:**
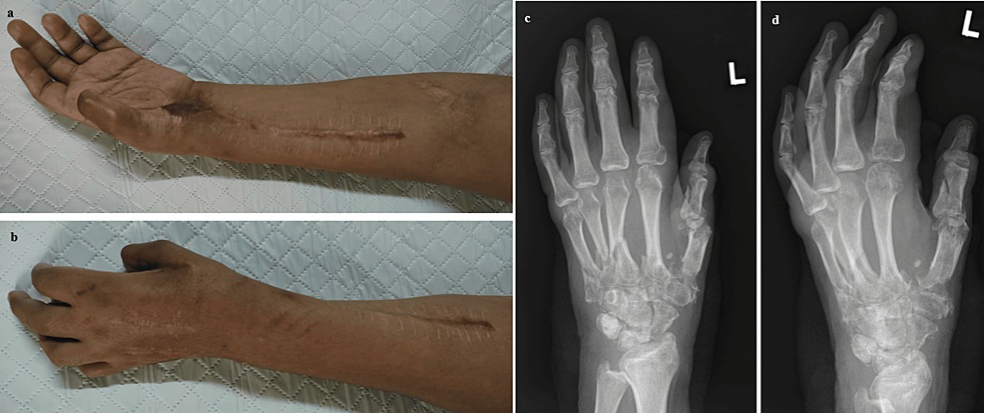
Clinical photos and left-hand radiographs at six months reveal significant improvement and positive progress in the patient's recovery journey. a-b) Clinical pictures reveal a fully healed wound on the left forearm, with the full-thickness skin graft applied over the left wrist flexor crease and thenar eminence displaying successful integration, all without any signs of contracture. c-d) Left-hand radiographs at six months show fracture callus formation with satisfactory alignment.

Upon inspecting the wound, it was observed that the forearm wound had healed well. Furthermore, the full-thickness skin graft applied over the left wrist flexor crease and thenar eminence exhibited successful uptake, with no signs of wound contracture observed. Follow-up hand radiographs revealed fracture callus formation with satisfactory alignment (Figure 7a-7d).

The recovery process underscored the significance of meticulous planning, including emergency interventions, surgical procedures, and continuous monitoring.

## Discussion

A crush injury of the hand is defined as a complex injury of the hand that involves multiple structures, including muscle, bone, skin, and neurovascular structures [1]. Comprehensive history-taking is crucial, encompassing details, such as the injury's mechanism, time of occurrence, and surrounding circumstances [1]. Lahiri et al. [12] proposed a guideline for the evaluation of hand injuries, outlining a comprehensive approach to assess various aspects, such as the mechanism of injury, clinical presentation, imaging studies, and treatment options. Tulipan et al. [11] proposed a novel classification system for open fractures of the hand, taking into consideration the unique factors specific to the hand that influence its risk of infection following an open fracture.

To mitigate systemic and renal complications associated with crush syndrome, early and robust fluid resuscitation is crucial [13]. Preferably, this should be initiated at the injury site before extrication [13]. A high clinical suspicion of compartment syndrome, especially in an insensate limb, in our case study, emphasizes the need for immediate surgical decompression and aggressive fluid resuscitation. This proactive approach can prevent complications, such as acute renal failure, reducing both mortality and morbidity.

Quantifying the extent of injuries immediately after trauma poses a challenge, complicating decision-making. Inadequate treatment can result in functional disability, while overly aggressive surgeries may increase morbidity and hand dysfunction. Therefore, optimizing treatment is paramount. During initial evaluation, distinguishing salvageable limbs from unsalvageable ones proves difficult [14]. To aid decision-making, scoring systems have been employed, such as the Mangled Extremity Severity Score (MESS), originally designed for severe lower limb injuries, with a score of seven or higher indicating amputation. Notably, the upper limb lacks a dedicated scoring system for amputation versus repair procedures, and the application of MESS in upper extremity cases lacks proper validation [14].

The primary objective of the initial surgery is to address dead tissues and debris, identify structures, achieve bony stabilization, and restore vascularity [15]. In contrast to lower limb trauma, where prostheses may at times yield superior outcomes compared to limb salvage, we emphasize prioritizing efforts to salvage upper extremities [16]. During the reconstruction of hand crush injuries, specific priorities must be established. Preserving the thumb, ideally up to the interphalangeal joint, is crucial, and having at least one or two fingers for pinching with the thumb is essential [17]. The reconstructed hand should exhibit robust and enduring soft tissue coverage while maintaining preserved sensation [17]. The success of the reconstruction is influenced by various factors, with early or immediate reconstruction generally yielding superior outcomes compared to delayed reconstruction [18]. Early and planned rehabilitation also plays a role in achieving favorable results.

In cases of closed BPIs without other immediate emergent issues, immediate surgical exploration and intervention may not be immediately necessary [19]. Recommendations include a thorough assessment of the situation, efficient pain management, and the initiation of rehabilitation [19]. Electromyography may be scheduled after three or four weeks, while CT/myelography or MRI is considered around six to eight weeks if denervation persists [20]. Surgical intervention may be contemplated after three to six months in instances of insufficient functional recovery or loss of neurological function [19]. The aim of conservative treatment is to maintain the extremity's range of motion, strengthen remaining functional muscles, protect denervated dermatomes, and effectively manage pain [17]. Our patient is displaying signs of recovery, including the restoration of elbow flexion, advancement in Tinel sign, and the presence of deep muscle aches.

In this unique case of a crush injury to the insensate hand, comprehensive and timely management is crucial. This includes early surgical intervention, a high index of clinical suspicion for acute compartment syndrome and crush syndrome, appropriate fluid resuscitation, and postoperative rehabilitation.

## Conclusions

The management of crush injuries of the hand demands a precise and early assessment, necessitating a systematic and individualized approach for each patient. Primary emergency surgery focuses on the removal of non-salvageable tissues, achieving skeletal stability, and revascularization. Subsequent surgeries aim to enhance hand function. This case, in conjunction with the discussion on crush syndrome and occult compartment syndrome, offers valuable insights into the intricate challenges and considerations involved in addressing severe crush injuries of the hand.

## References

[1] Wang W, Schindelar L, Tosti R (2020). An updated review on the emergency management of the mangled upper extremity. Curr Trauma Rep.

[2] Greaves I, Porter K, Smith JE (2003). Consensus statement on the early management of crush injury and prevention of crush syndrome. J R Army Med Corps.

[3] Smith J, Greaves I (2003). Crush injury and crush syndrome: a review. J Trauma.

[4] Malinoski D, Slater M, Mullins R (2004). Crush injury and rhabdomyolysis. Crit Care Clin.

[5] Rios-Alba T, Ahn J (2015). A swollen hand with blisters: a case of compartment syndrome in a child. Pediatr Emerg Care.

[6] Volkmann R (1881). The ischemic muscular paralysis and trauma [Article in German]. Zentralblatt fur Chirurgie.

[7] Mabvuure NT, Malahias M, Hindocha S, Khan W, Juma A (2012). Acute compartment syndrome of the limbs: current concepts and management. Open Orthop J.

[8] Wright JG, Bogoch ER, Hastings DE (1989). The 'occult' compartment syndrome. J Trauma.

[9] Schmidt AH (2016). Acute compartment syndrome. Orthop Clin North Am.

[10] Janzing H, Broos P (2001). Routine monitoring of compartment pressure in patients with tibial fractures: beware of overtreatment!. Injury.

[11] Tulipan JE, Ilyas AM (2018). Open fractures of the hand: review of pathogenesis and introduction of a new classification system. Hand Clin.

[12] Lahiri A (2020). Guidelines for management of crush injuries of the hand. J Clin Orthop Trauma.

[13] Odeh M (1991). The role of reperfusion-induced injury in the pathogenesis of the crush syndrome. N Engl J Med.

[14] Togawa S, Yamami N, Nakayama H, Mano Y, Ikegami K, Ozeki S (2005). The validity of the mangled extremity severity score in the assessment of upper limb injuries. J Bone Joint Surg Br.

[15] Alphonsus CK (2011). Principles in the management of a mangled hand. Indian J Plast Surg.

[16] Gautam P, Gyawali S, Mainali P, Niraula H, Shrestha JM, Lohani I (2023). Mangled right hand: a case report. Int J Surg Case Rep.

[17] Bernstein ML, Chung KC (2007). Early management of the mangled upper extremity. Injury.

[18] Kline DG (1989). Civilian gunshot wounds to the brachial plexus. J Neurosurg.

[19] Wade RG, Takwoingi Y, Wormald JC, Ridgway JP, Tanner S, Rankine JJ, Bourke G (2019). MRI for detecting root avulsions in traumatic adult brachial plexus injuries: a systematic review and meta-analysis of diagnostic accuracy. Radiology.

[20] Dubuisson A, Kline D (2002). Brachial plexus injury: a survey of 100 consecutive cases from a single service. Neurosurg.

